# Professional Etiquette1Read before the Passaic County Veterinary Medical Association, at Paterson, N. J., October 7, 1902.

**Published:** 1902-11

**Authors:** J. Payne Lowe

**Affiliations:** Passaic, N. J.


### PROFESSIONAL ETIQUETTE.[1]

By J. Payne Lowe, D.V.S.
PASSAIC, N. J.

The subject assigned to me is an important one to each of us individually and to us collectively as members of the veterinary profession; therefore it is worthy of the consideration of this Association.

It is only necessary for me to make mention of the rapid advancement our profession is now making in New Jersey; of her strong State Association and the good work it is doing; of the law passed at the last Legislature regulating the practice of veterinary medicine, surgery, and dentistry; of the appointment by the Governor of a State Board of Veterinary Medical Examiners; of the recognition our profession and associations are receiving from other professions and kindred organizations, and now of the organization in this county of the first county society in the State.

In view of all this, more is to be expected from us. Is it not important that each of us, at all times, live up to a proper standard

[1] Read before the Passaic County Veterinary Medical Association, at Paterson, N. J., October 7, 1902.

of professional etiquette? Etiquette has been defined as the formalities or usages required by the customs of polite society or professional intercourse. It is not alone sufficient for the veterinarian of to-day to have the proper qualifications, both theoretical and practical, but he must observe the rules laid down by professional etiquette for his own individual benefit and for the welfare of his profession.

Every individual on entering the veterinary profession is entitled to all its privileges and immunities, and it then becomes his obligation to exert his best abilities to maintain its dignity and honor and to aid in advancing it as a science.

We have in Passaic County, I believe, about twenty licensed veterinarians, all more or less actively engaged in practice and coming into contact, directly or indirectly, more or less frequently. It is necessary for each of us to plainly understand where our duties leave off and where those of the next man begin. Under the law we all have, whether graduates or non-graduates, the same legal right to practice, and, therefore, all are entitled to the same consideration.

Often an owner has an animal taken acutely sick or severely injured, and he will summon a veterinarian; but perhaps the doctor is out, and so he hurriedly calls another who happens to be in, and he responds to the call at once. After a short time the first called doctor returns, and he, too, responds to the call, only to find some one there ahead of him. Under usual circumstances the case belongs to the man arriving first; so after a few brief words with the owner and veterinarian in charge he should bid them good-day and retire, as it is undignified for him to remain, and perhaps his presence is annoying to the attending veterinarian, who at this time is busy and does not care to be interrupted. But there are duties of etiquette for the attending veterinarian to perform. He should, if possible, leave his patient a moment and greet his brother practitioner and make him feel at ease; and if the owner has acted rashly in calling upon both at or about the same time, he should explain to him that he has interrupted a practitioner in his routine work. Further than this, he should sustain his brother in collecting a fee for his visit, unless, of course, he has been tardy in responding to the call, in which case he should not expect a fee. If this is carried out with tact in practice it will have the effect of educating the laity and prevent much annoyance.

Again, sometimes two veterinarians are treating cases for their respective clients in a boarding-stable. Certainly neither one of them should so far lower his dignity as to even attempt to examine or express an opinion of the case in charge of his brother practitioner.

Sometimes a case under the treatment of a qualified practitioner does not show the improvement that the owner from his standpoint thinks it should—it is only natural for him to do a lot of thinking—and here is where a lot of tact is required on the part of the veterinarian in order to hold the confidence of his client. Different clients will act differently under such circumstances. One may, without the knowledge of the attending veterinarian, call in another practitioner. As a rule, if the second veterinarian so called in has his eyes open, and has the desire, he will discover what is going on. Knowing this, he should call upon the owner for an explanation, and should make no examination or give any opinion or treatment until he knows that the other veterinarian has been properly dismissed.

If a consultation is desired by the owner it is good sense for the practitioner to at once accede to it. In consultation the first object should be for the good of the patient, in the interest of the owner. Perhaps the case is complicated or obscure in its nature, and a consultation with a man of experience and judgment will enlighten the attending veterinarian; or, again, it may be held for the protection of the attending veterinarian—viz., in a necessarily fatal case, where the owner thinks possibly something more might be done to save the life of his animal—and here the consulting veterinarian can restore the owner's confidence in his doctor; or, again, a consultation may be held between several veterinarians, with the object in view of dividing the responsibility, as in the case of a valuable animal that may have to undergo an operation; or, again, it may be advisable to hold a consultation where litigation is apt to follow, in order to have sufficient expert evidence to satisfy the court of the conditions that existed.

However, whatever the object of the consultation, no rivalship or jealousy should be indulged in; and candor, with all due respect, should be exercised toward the veterinarian having charge of the case. The consulting veterinarian should, so far as he can, conscientiously sustain the attending veterinarian. After the history of the case is given and an examination of the patient made, they should retire for deliberation. Theoretical discussions should, as far as possible, be avoided as causing perplexity and loss of time.

The conclusion arrived at should be given to the owner by the attending veterinarian, in the presence of all concerned; and then, as a matter of courtesy to the consulting veterinarian, he may be asked to give the owner, in his own words, his opinion of the case. If the attending veterinarian has made a mistake in his case, and it is apparent, he should not take it to heart if his brother points out his error. As long as it is done in a kind way, and he is not unnecessarily exposed, he should feel that he has gained by the experience given him; and, above all, young men in the profession should show due respect to those older and with more experience. Perhaps on some rare occasion the two veterinarians cannot agree—a thing to be regretted. If the difference of opinion is of any practical importance they should call in the third man and agree to abide by his opinion.

I might go on almost indefinitely expatiating on the subject of etiquette, but will only add that as professional men we should observe the rules of etiquette in a liberal way, not only among ourselves as veterinarians, but among all scientific men and to people in general.

I do not wish it to be thought that this is intended as a criticism toward the practitioners of Passaic County, and I have no fault to find, for it has been my pleasure and profit to meet many of you in a professional way, and I can truthfully say that I have always been treated with perhaps more consideration than I deserved. However, we can all improve, and if the few salient points I have tried to bring out in my paper are actually lived up to in practice by the veterinarians of this county it will elevate us and our profession in the eyes and minds of the community, and we will reap the benefit thereof.

